# Association Analysis of ARHGEF37 Gene Polymorphisms with Growth Traits in Pigs

**DOI:** 10.3390/ani16213411

**Published:** 2026-10-29

**Authors:** Leshan Guo, Xuguang Yang, Junyan Bai, Xiangdong Ding, Xin Qi, Xinjian Yang, Xuewen Gong, Yang Liu, Renjie Liang

**Affiliations:** 1 College of Animal Science and Technology, Henan University of Science and Technology, Luoyang 471023, China; wuli_shan258@163.com (L.G.); m15236505751@163.com (X.Y.); lrjdsp@163.com (R.L.); 2 College of Animal Science and Technology, China Agricultural University, Beijing 100193, China; xding@cau.edu.cn; 3 Xinjiang Aksu Joint Research Institute of China Agricultural University, Aksu 843000, China; yangxj_001@163.com; 4 Aksu Xingjiang Muge Food Co., Ltd., Aksu 843000, China; 15292302025@163.com (X.Q.); ha139@163.com (X.G.); liuyoung8023@163.com (Y.L.); 5 Aksu Science and Technology Innovation Center, Aksu 843000, China

**Keywords:** Large White pig, Landrace pig, SNP, growth traits

## Abstract

Growth traits are key economic indicators in pig breeding, and identifying molecular markers associated with body weight, backfat thickness, loin eye depth, body length, chest girth, and chest width is of great importance for improving the efficiency of marker-assisted selection (MAS). In this study, 400 French Large White sows and 40 French Landrace sows were used as experimental populations. Single nucleotide polymorphisms (SNPs) within the *ARHGEF37* gene were genotyped using PCR amplification and sequencing. Genotype frequencies, allele frequencies, and genetic diversity parameters were systematically analyzed, and associations between different genotypes, haplotypes, and growth traits were evaluated using one-way analysis of variance (ANOVA). Several variants showed nominal associations with growth traits in the French Large White population, although none remained significant after FDR correction; *ARHGEF37* is therefore proposed as a candidate gene requiring validation in larger populations. Additionally, haplotype analysis revealed that certain haplotype combinations exhibited synergistic improvements in economically important traits including body weight, backfat thickness, and chest circumference, providing potential genetic targets for marker-assisted selection.

## Simple Summary

How fast pigs grow and how much feed they need are key factors for pig farmers, because they directly affect production costs and income. Breeders usually improve growth by measuring traits such as body weight, backfat thickness, and body size, but this takes time and requires raising animals to a certain age. Finding small natural differences in the DNA that are linked with better growth could help breeders choose the best animals earlier and more accurately. In this study, we looked at one gene, called *ARHGEF37*, in 400 Large White sows and 40 Landrace sows from commercial herds. We identified several DNA variations in this gene and tested whether they were related to growth traits. In Large White pigs, some variations showed nominal associations with body weight, backfat thickness, and chest size, and certain combinations of variations were connected with better overall growth, but these require confirmation in larger populations. These findings suggest that *ARHGEF37* is a candidate gene for growth traits that merits further study. These results may help breeders develop DNA-based selection tools, making pig production more efficient and supporting more sustainable farming.

## 1. Introduction

Growth traits are among the most important economic traits in pig breeding and directly affect feed conversion efficiency, the production cycle, and farming profitability. Body weight, average daily gain, body length, backfat thickness, and loin eye area have become core parameters for evaluating production performance in modern pig breeding. As conventional phenotypic selection approaches its limit, marker-assisted selection (MAS) has become an important strategy for improving selection efficiency and advancing the genetic improvement of pigs.

Single-nucleotide polymorphisms (SNPs) and small insertions and deletions (InDels) are the most common types of genetic variation in animal genomes. They play important roles in regulating growth and development, reproductive performance, meat quality traits, and disease resistance [1,2]. In recent years, whole-genome resequencing technology has developed rapidly. Its broad coverage, high detection accuracy, and high throughput provide strong support for efficiently identifying functional mutations and elucidating the genetic basis of complex economic traits [3]. Previous studies have shown that SNPs in several candidate genes are closely associated with growth performance in pigs. For example, polymorphisms in PTER and CALB2 were significantly associated with growth traits in American Large White pigs [4]. Multiple SNPs and haplotypes in ABCC10 were significantly associated with growth traits and the maternal index in Danish Large White pigs [5]. Polymorphisms in IRS1 were closely associated with age at 100 kg body weight, average daily gain, and loin eye area in Large White pigs [6]. These findings indicate that identifying functional variants associated with growth traits is important for improving pig breeding efficiency.

*ARHGEF37* (Rho guanine nucleotide exchange factor 37) belongs to the Rho GTPase signaling pathway, which plays critical roles in cell proliferation, differentiation, and skeletal muscle development. As a guanine nucleotide exchange factor, *ARHGEF37* activates Rho family GTPases, which are key regulators of cytoskeletal dynamics, cell migration, and growth signaling pathways. Previous studies have shown that Rho GTPase signaling is essential for myoblast fusion, muscle fiber growth, and overall skeletal muscle development in mammals, making genes in this pathway biologically plausible candidates for growth trait variation [7]. Based on this biological rationale, we selected *ARHGEF37* as a candidate gene for investigating genetic variation underlying growth traits in pigs.

French Large White and French Landrace pigs are important lean-type breeds used in commercial pig production in China. They exhibit rapid growth, a high lean meat percentage, and favorable reproductive performance. They are also important genetic resources in nucleus breeding systems. Therefore, clarifying the genetic effects of candidate gene variants in these two breeds is of practical importance for improving molecular breeding systems.

Accordingly, this study used nucleus breeding populations of French Large White and French Landrace pigs. Candidate SNPs in *ARHGEF37* were genotyped, and their population genetic characteristics and associations with growth traits were analyzed. Their potential use as markers for MAS was also evaluated. This study aimed to provide a theoretical basis and candidate molecular markers for precision breeding and genetic improvement of French Large White and French Landrace pigs.

## 2. Materials and Methods

### 2.1. Animals and Phenotype Data

French Large White and French Landrace sows raised by Aksu Hongsheng Animal Husbandry Co., Ltd. (Aksu, China) were used as the experimental animals. They are hereafter referred to as Large White and Landrace pigs, respectively. Hair follicle samples and phenotypic records for six key growth traits were collected from 400 Large White pigs and 40 Landrace pigs. All pigs were housed in standard commercial facilities with ad libitum access to feed and water. They were maintained under uniform management conditions following standard commercial breeding practices, with controlled ventilation, temperature regulation, and regular health monitoring. The traits included age at target body weight, body length, chest girth, chest width, backfat thickness (BF), and loin muscle depth (LMD). Measurements were performed according to NY/T 820-2004 [8]. This study was approved by the Animal Experiment Ethics Committee of Henan University of Science and Technology (approval no. HAUST#2025-2-15). All animal procedures were conducted in accordance with the Guidelines for the Care and Use of Laboratory Animals in China. Sample collection involved only non-invasive hair follicle sampling from live animals during routine management. All animals were handled with care to minimize stress during sample collection.

Due to budget constraints, a subset of animals was selected for genotyping from larger phenotyped populations. For Large White pigs, 182 animals were randomly selected from 400 phenotyped animals for DNA extraction and genotyping. For Landrace pigs, 36 animals were randomly selected from 40 phenotyped animals. The smaller Landrace sample size reflects both the smaller available population at the farm and budget limitations for genotyping. Animals were randomly selected to ensure representative sampling across the phenotypic range of each trait. All selected samples achieved a 100% genotyping call rate across all loci, indicating high-quality DNA and successful genotyping for all individuals.

### 2.2. DNA Extraction and Genotype Data

The *ARHGEF37* gene (NCBI Gene ID: 100525410) is located on pig chromosome 4 (Sus scrofa 11.1 assembly). Exon 8 was selected as the target region because it encodes a portion of the Dbl homology (DH) domain, which is critical for the guanine nucleotide exchange activity of *ARHGEF37*. Functional variants in this region may directly affect protein function and downstream signaling pathways related to cell growth and proliferation. Genomic DNA was extracted from pig hair follicles using the DN25 ultramicro/clinical genomic DNA rapid extraction kit (Aidlab Biotechnologies Co., Ltd., Beijing, China) following the manufacturer’s instructions. Specific primers targeting exon 8 of the *ARHGEF37* gene were designed with Primer 5.0 software. The forward primer F (5′–3′) was TTTGACGACCACCCGATAC and the reverse primer R (5′–3′) was CTGCTGCGTTTGAGTCCC, with an expected amplicon length of 857 bp. PCR was performed in a 10 μL reaction mixture containing 0.3 μL of each primer, 5 μL of 2× Taq PCR Master Mix (Tiangen Biotech [Beijing] Co., Ltd., Beijing, China), 0.6 μL of genomic DNA template, and 3.8 μL of ddH_2_O. The cycling program consisted of initial denaturation at 94 °C for 2 min, followed by 30 cycles of denaturation at 94 °C for 30 s, annealing at 59 °C for 30 s, and extension at 72 °C for 30 s, then a final extension at 72 °C for 2 min and holding at 4 °C. The PCR products were separated by electrophoresis on a 1.5% agarose gel (Biowest, Nuillé-sur-Vicoin, France) at a constant voltage of 110 V for 30 min, and the target bands were visualized with a gel imaging system. Genomic DNA from 182 Large White and 36 Landrace samples was used for PCR amplification. The hair-follicle samples analysed in this study were provided by Aksu Hongsheng Animal Husbandry Co., Ltd. (Aksu, China). Qualified PCR products were directly sequenced, and sequence alignment was performed with DNAMAN. PCR amplification was performed using primers designed to amplify the entire *ARHGEF37* exon 8 region (approximately 600 bp) as a single long-range fragment, rather than amplifying individual SNP sites separately. This long-range amplification strategy was essential for maintaining the physical linkage of alleles on the same chromosome, thereby enabling direct experimental determination of haplotype phase from Sanger sequencing chromatograms without requiring computational phase inference.

### 2.3. Genetic Diversity Analysis

SNPs were identified from the sequencing chromatograms, and each SNP and its genotypes were named according to the corresponding coding-sequence position and nucleotide substitution. Individual haplotypes were determined from the sequencing chromatograms. Allele and genotype frequencies at each locus were calculated with Excel, and Hardy–Weinberg equilibrium was tested (*p* < 0.01 indicated deviation from equilibrium). Hardy–Weinberg equilibrium (HWE) was tested for each SNP in both breeds using chi-square tests. Given the multiple SNPs tested, we used a more stringent significance threshold of *p* < 0.01 (instead of *p* < 0.05) to identify SNPs deviating from HWE, which could indicate genotyping errors or strong population substructure. The polymorphism information content (PIC), heterozygosity (He), homozygosity (Ho), and the effective number of alleles (Ne) were calculated using the online Genetic Diversity Index Calculator (msrcall.com/Gdicall.aspx). Linkage Disequilibrium Analysis: Pairwise linkage disequilibrium (LD) between all SNPs was calculated using PLINK 1.9. Two LD measures were computed: (1) r^2^ (squared correlation coefficient): Measures the correlation between alleles at two loci. Values range from 0 (complete independence) to 1 (complete LD). r^2^ represents the proportion of variance at one locus explained by the other locus. r^2^ > 0.8 is conventionally considered strong LD. (2) D’ (standardized disequilibrium coefficient): Measures the deviation from linkage equilibrium relative to the maximum possible deviation given the allele frequencies. Values range from 0 to 1, with D’ = 1 indicating complete LD (no historical recombination between the loci). LD analysis was performed separately for Large White and Landrace populations to assess the degree of redundancy among SNPs and to aid in interpretation of association results.

### 2.4. Statistical Models

Associations of SNP genotypes and haplotypes with growth traits were analyzed using analysis of variance under the general linear model (GLM) in SPSS 26. The model was as follows:

$$\;Y_{ik}=\mu +F_{i}+D_{ik}+e_{ik}$$

where $Y_{ik}$ denotes the i th genotype and the k th individual’s phenotypic value, including body weight, backfat thickness, loin muscle depth, body length, chest girth, and chest width; μ is the overall mean; $F_{i}$ represents the fixed genotype effect, such as II, ID, or DD; in the haplotype analysis, $F_{i}$ represents the fixed haplotype effect, such as H1, H2, or H3; $D_{ik}$ is the age at measurement; $e_{ik}$ is the random error, which was assumed to follow a normal distribution. Marginal means for each genotype were calculated using the estimated marginal means function, and pairwise comparisons were conducted using Duncan’s multiple-range test. For comparisons among genotypes at the same locus, identical lowercase letters indicate nonsignificant differences, whereas different lowercase letters indicate significant differences (*p* < 0.05).

Due to data availability limitations, other potential covariates such as pedigree structure, birth batch effects, pen effects, and farm environmental factors were not available and could not be included in the model. All animals were raised at the same commercial farm under standardized management conditions, which partially controls for environmental variation. However, we acknowledge that unmeasured environmental factors and population structure could influence the results.

### 2.5. Statistical Analysis

To account for multiple comparisons across SNPs, traits, and breeds, we applied false discovery rate (FDR) correction using the Benjamini–Hochberg procedure. The total number of comparisons planned included SNP–trait associations (9 SNPs × 6 traits in Large White + 8 SNPs × 6 traits in Landrace after filtering) and haplotype–trait associations (7 haplotypes × 6 traits in Large White), totaling 144 nominal comparisons. After excluding rare genotypes and haplotypes with *n* < 5, as well as comparisons for which fewer than two valid groups remained, 78 comparisons were actually performed and subjected to FDR (Benjamini–Hochberg) correction. We calculated FDR-adjusted *q*-values for these 78 tests. Associations were considered statistically significant at *q* < 0.05 after FDR correction. To ensure statistical reliability, genotypes or haplotypes with fewer than 5 individuals were excluded from association analysis. Haplotype phase was directly determined by Sanger sequencing of long-range PCR products encompassing all polymorphic sites within *ARHGEF37* exon 8. Each amplicon covered approximately 600 bp containing all 9-10 SNPs. For individuals heterozygous at multiple sites, phase was unambiguously resolved from sequencing chromatograms without requiring computational inference, trio data, or cloning. This direct experimental approach provides validated haplotype phase for all individuals with a 100% call rate. This threshold was chosen based on established practices in genetic association studies to ensure adequate sample size for reliable effect estimation and to prevent inflated Type I error rates. Values are presented as mean ± standard error. FDR correction was applied using the Benjamini–Hochberg method for multiple comparisons.

## 3. Results

### 3.1. PCR Amplification

**Figure 1:**
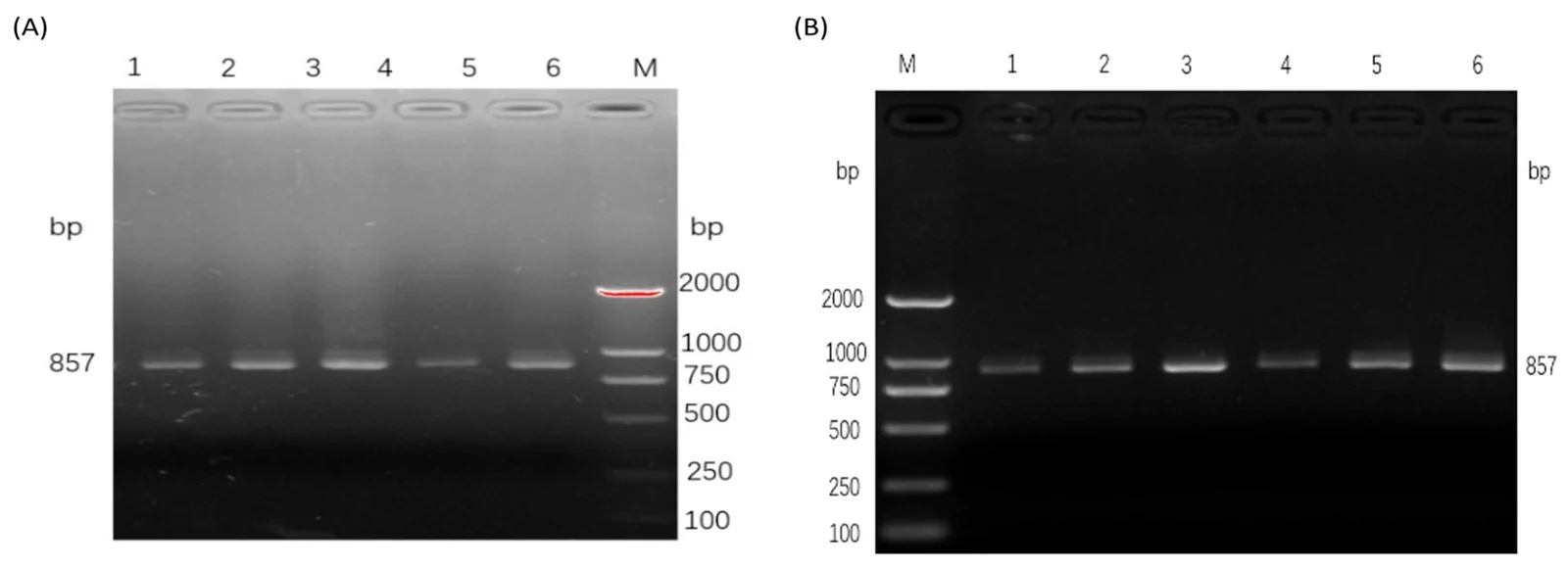
PCR amplification of *ARHGEF37*. (**A**) in the Large White population. (**B**) in the Landrace population. The red line in (**A**) indicates the position of the expected 857-bp target band.

Specific amplification of *ARHGEF37* was observed in both French Large White and Landrace populations. Agarose gel electrophoresis (Figure 1A,B) yielded a single clear 857-bp band consistent with the expected fragment size, which was suitable for subsequent sequencing analysis.

### 3.2. Analysis of Sequencing Results

**Figure 2:**
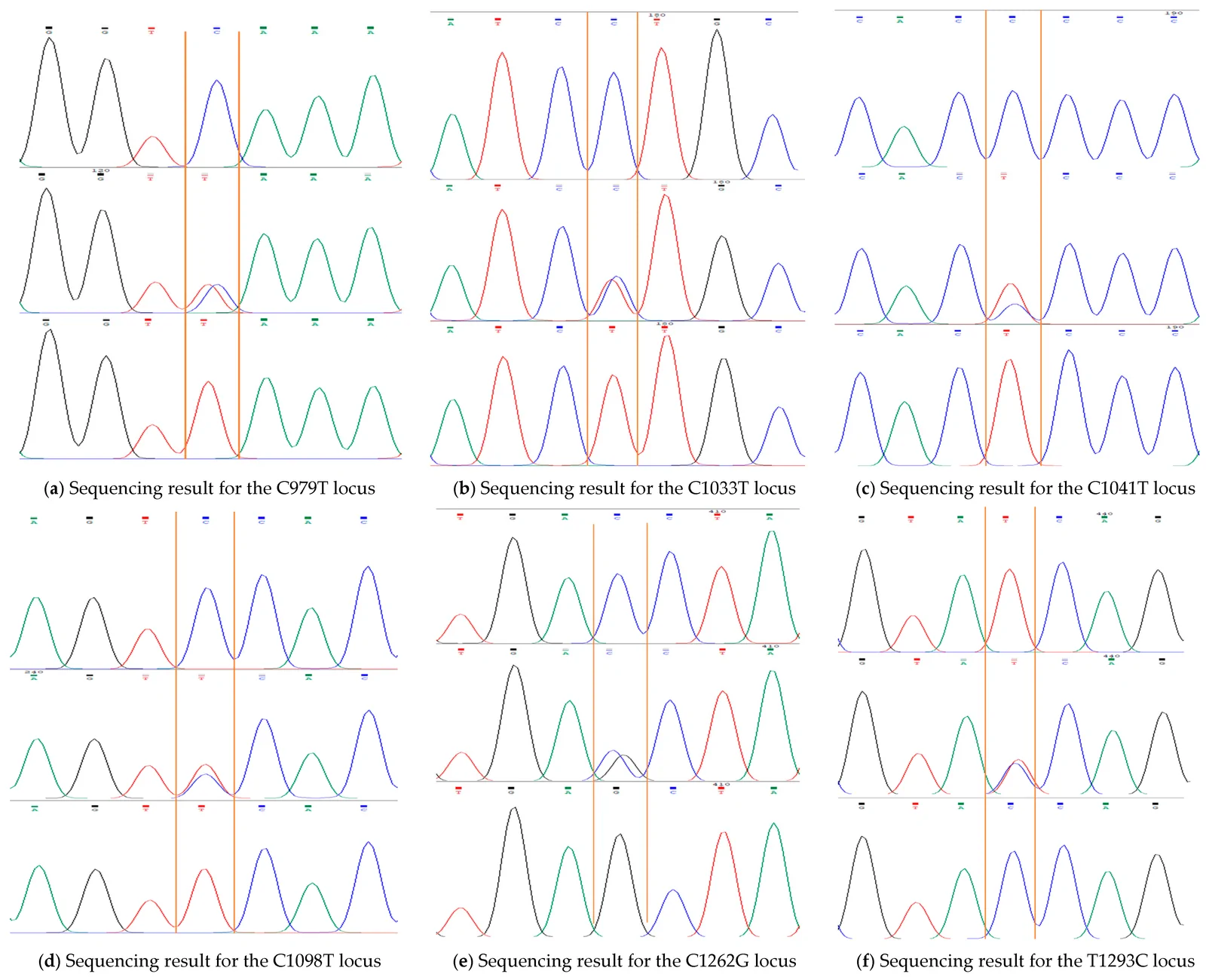

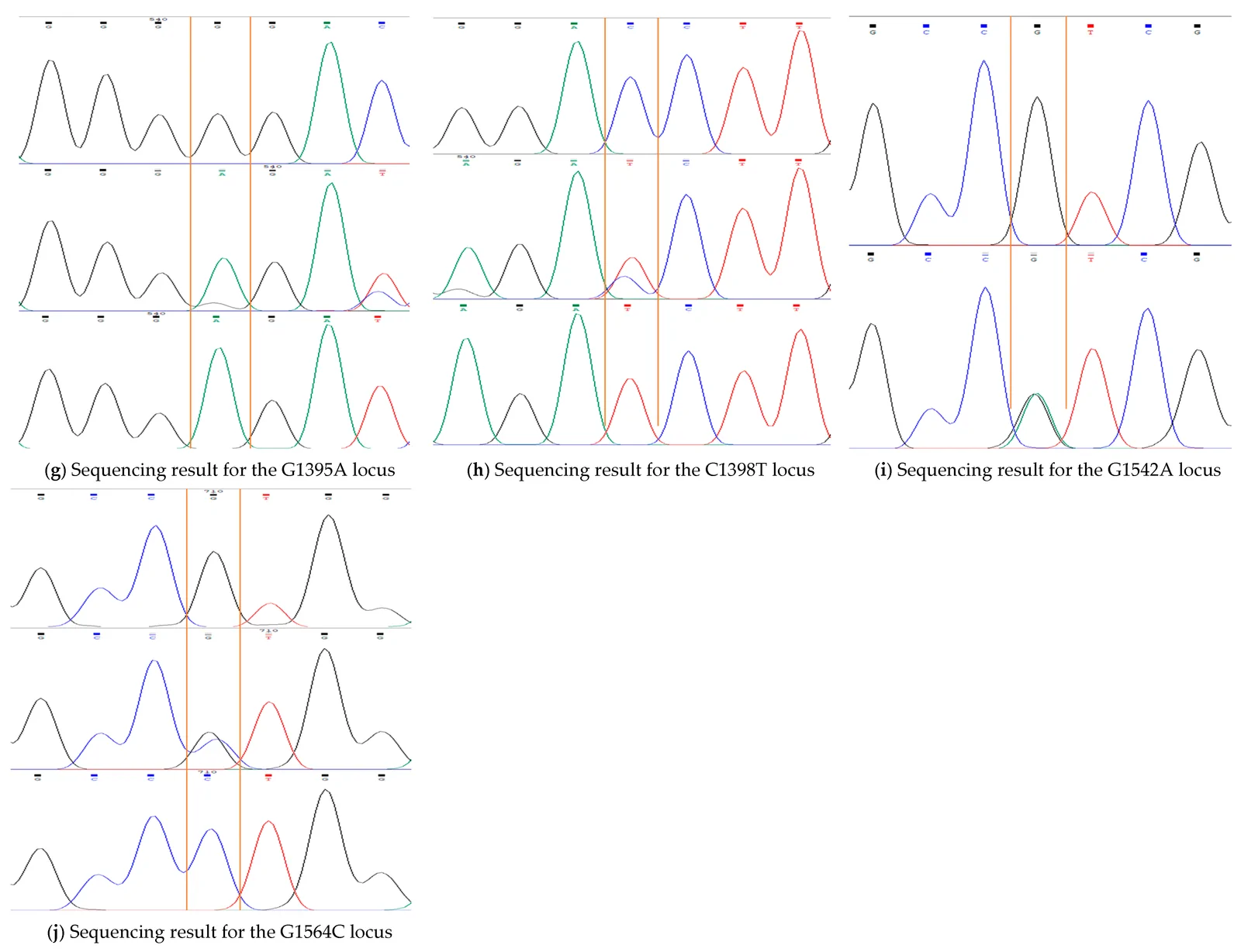
Ten SNPs in the *ARHGEF37* gene. (**a**) C979T. (**b**) C1033T. (**c**) C1041T. (**d**) C1098T. (**e**) C1262G. (**f**) T1293C. (**g**) G1395A. (**h**) C1398T. (**i**) G1542A. (**j**) G1564C. The colored vertical lines indicate the positions of the SNP loci.

Sequencing of PCR products from Large White and Landrace pigs identified 10 SNPs in exon 8 of *ARHGEF37* (Figure 2): C to T at position 979 of the CDS (C979T; Figure 2a), C to T at 1033 (C1033T; Figure 2b), C to T at 1041 (C1041T; Figure 2c), C to T at 1098 (C1098T; Figure 2d), C to G at 1262 (C1262G; Figure 2e), T to C at 1293 (T1293C; Figure 2f), G to A at 1395 (G1395A; Figure 2g), C to T at 1398 (C1398T; Figure 2h), G to A at 1542 (G1542A; Figure 2i), and G to C at 1564 (G1564C; Figure 2j).

### 3.3. Allele and Genotype Frequencies of ARHGEF37 SNPs

In the Large White population, nine SNPs were detected in *ARHGEF37*: C979T, C1033T, C1041T, C1098T, C1262G, T1293C, G1395A, C1398T, and G1564C. Three genotypes were identified at each of these nine SNPs; the distributions of genotype and allele frequencies are shown in Table 1. In the Landrace population, ten SNPs were detected in *ARHGEF37*: C979T, C1033T, C1041T, C1098T, C1262G, T1293C, G1395A, C1398T, G1542A, and G1564C. Except for C1098T and C1262G, at which three genotypes were detected, only two genotypes were observed at each of the other eight loci. The genotype and allele frequency distributions are shown in Table 1.

### 3.4. Genetic Diversity Analysis of ARHGEF37 SNPs

Under a stringent threshold (*p* < 0.01), most SNPs were in Hardy–Weinberg equilibrium in both breeds. The only deviations were G1564C in Large White (*p* = 0.0072) and C1262G in Landrace (*p* < 0.05); these two loci should therefore be interpreted with caution.

In the Large White population, Table 2 showed that all loci except G1564C (*p* = 0.0072) were in Hardy–Weinberg equilibrium (*p* > 0.01), indicating a relatively stable genetic structure. Homozygosity ranged from 0.55 to 0.77, indicating that homozygotes accounted for 55–77% of the population. T1293C had the highest homozygosity (0.77), whereas C1098T and C1262G had the lowest (0.55). At most loci, the effective number of alleles ranged from 1.63 to 1.82, close to the theoretical maximum of 2, indicating a relatively balanced allele distribution. Except for T1293C, which showed low polymorphism (PIC = 0.20), all other loci showed moderate polymorphism (PIC = 0.31–0.35), indicating relatively rich genetic diversity suitable for genetic diversity evaluation and association analysis. In the Landrace population, C979T, G1395A, and C1398T had the highest homozygosity, all reaching 0.95, indicating that these loci were nearly completely homozygous. C1098T had the lowest homozygosity (0.69). Only C1262G deviated from Hardy–Weinberg equilibrium (*p* < 0.05); the remaining loci were in equilibrium. Only C1041T and C1098T showed moderate polymorphism (PIC = 0.25–0.26) and relatively rich genetic diversity. The remaining loci showed low polymorphism (PIC ≤ 0.24), indicating low overall genetic diversity.

Linkage Disequilibrium Analysis: Pairwise linkage disequilibrium (LD) analysis revealed varying degrees of correlation among the SNPs within *ARHGEF37* exon 8 (complete LD results in Supplementary Table S2). Large White Population: Strong LD relationships were observed between several SNP pairs: Complete LD (r^2^ = 1.00, perfect correlation): C1033T and C1041T: r^2^ = 1.00, D’ = 1.00. These two SNPs are in perfect linkage disequilibrium, meaning they are completely co-inherited in this population. Their individual effects cannot be distinguished, and they provide redundant information. Any association detected for one SNP applies equally to the other. High LD (r^2^ > 0.90): G1395A and C1398T: r^2^ = 0.95, D’ = 0.98. These SNPs show near-perfect LD and are almost always inherited together. The strong LD between C1033T-C1041T and G1395A-C1398T means that associations detected for these SNP pairs likely reflect the same underlying genetic signal rather than independent effects. This redundancy should be considered when interpreting the number of independent associations.

### 3.5. Differences in Growth Traits Among ARHGEF37 SNP Genotypes in the Pig Populations

Growth-trait association analyses were conducted separately in the Large White and Landrace populations. All analyses were performed using SPSS 26, and growth-trait data are presented as means ± standard errors. For comparisons among genotypes at the same locus, identical lowercase letters indicate nonsignificant differences, whereas different letters indicate significant differences (*p* < 0.05). In addition, only one individual was detected for certain genotypes at some loci. Because the sample size was too small to obtain reliable estimates of genotype-specific trait means or perform valid between-group statistical comparisons, these genotypes were excluded from the association model. Specifically, these were the CT genotype at C979T, the TT genotype at C1098T, and the CG and GG genotypes at C1262G in the Landrace population. Association analysis between *ARHGEF37* SNP genotypes and growth traits in Large White (*n* = 182) and Landrace (*n* = 36) pigs was conducted.

In the Large White population (Table 3), C979T, C1098T, C1262G, and T1293C were not significantly associated with any measured growth trait. At C1033T and C1041T, individuals with the TT genotype had significantly greater body weight than those with the CC and CT genotypes (*p* < 0.05). At G1395A, individuals with the AA genotype had significantly greater body weight than those with the GA and GG genotypes, and their chest girth was also significantly greater than that of GA individuals (*p* < 0.05). At C1398T, TT individuals had significantly greater body weight than CC and CT individuals (*p* < 0.05), and their chest girth was also significantly greater than that of CT individuals (*p* < 0.05). At G1564C, CC individuals had significantly greater body weight than GC individuals (*p* < 0.05). In the Landrace population (Table 4), C979T was not significantly associated with any growth trait. C1262G was not statistically analyzed. At C1033T, C1041T, and C1098T, CT individuals had significantly greater body weight and chest girth than CC individuals (*p* < 0.05). At T1293C, TC individuals had significantly greater body weight and chest girth than TT individuals (*p* < 0.05). At G1395A, GA individuals had significantly greater chest girth than GG individuals (*p* < 0.05). At C1398T, CT individuals had significantly greater chest girth than CC individuals (*p* < 0.05). At G1542A, GA individuals had significantly greater backfat thickness than GG individuals (*p* < 0.05). At G1564C, GC individuals had significantly greater body weight than GG individuals (*p* < 0.05). All *p* values reported above are uncorrected and did not survive FDR correction.

Statistical Significance After FDR Correction. After applying stringent FDR correction for 78 multiple comparisons (actual tests completed), none of the associations reached the statistical significance threshold (*q* < 0.05). However, several SNPs showed nominally significant associations with uncorrected *p* < 0.05. The most promising association was observed for G1564C with body weight in Landrace (*p* = 0.0038, *q* = 0.30), which showed nominal significance before correction but did not survive FDR adjustment. These nominally significant associations are reported in Supplementary Table S1, but should be interpreted cautiously as they did not survive correction for multiple testing.

### 3.6. Differences in Growth Traits Among ARHGEF37 Haplotypes

Growth-trait association analyses were conducted separately in the Large White and Landrace populations. All analyses were performed using SPSS 26, and growth-trait data are presented as means ± standard errors. For comparisons among haplotypes, identical lowercase letters indicate nonsignificant differences, whereas different letters indicate significant differences (*p* < 0.05). In addition, only one individual was detected for certain haplotypes. Because the sample size was too small to obtain reliable estimates of haplotype-specific trait means or perform valid between-group statistical comparisons, these haplotypes were excluded from the association model, as shown in Table 5.

Haplotype phase was successfully determined for all genotyped individuals using direct long-range PCR sequencing, achieving a 100% call rate with no phase ambiguity in both Large White (*n* = 182) and Landrace (*n* = 36) populations. This experimental approach provided validated haplotype phase without computational inference. To ensure statistical reliability and prevent inflated Type I error rates, genotypes and haplotypes with sample sizes less than 5 (*n* < 5) were excluded from association analysis. In Large White pigs, all SNP genotypes met this criterion (*n* ≥ 5) and were retained for analysis. However, 12 rare haplotypes with *n* < 5 were excluded, retaining 7 haplotypes that represented 128 of 182 individuals (70.3%). The retained haplotypes with *n* ≥ 5 were: H4 (CCCCCTGCG, *n* = 77), H19 (TTTTGTATC, *n* = 19), H1 (CCCCCCGCG, *n* = 8), H10 (CCTTGTATC, *n* = 7), H6 (CCCCGTGCG, *n* = 7), H3 (CCCCCTATG, *n* = 5), and H9 (CCTCGTATG, *n* = 5). In Landrace pigs, four SNP genotypes with insufficient sample sizes were excluded from association analysis: C979T-CT (*n* = 2), C1098T-TT (*n* = 1), C1262G-CG (*n* = 1), and C1262G-GG (*n* = 1). These genotypes are shown in the tables for completeness but were not included in statistical testing. Additionally, haplotype association analysis could not be performed in Landrace pigs due to limited haplotype diversity, with only one haplotype meeting the *n* ≥ 5 criterion while other haplotypes had sample sizes ranging from 1 to 3 individuals (Table 5). Genotyping of the Landrace population (*n* = 36) based on ten SNPs identified three main haplotypes accounting for 28 of the 36 individuals: H1 (CCCCCTGCGG, *n* = 23), H2 (CCTCCTGCGG, *n* = 3), and H3 (CTTTCCGCGG, *n* = 2). Because only H1 met the minimum sample size criterion (*n* ≥ 5), no valid between-haplotype comparison could be performed for the Landrace population. For completeness, all haplotypes are listed in Table 5; statistical comparisons were restricted to those with *n* ≥ 5.

## 4. Discussion

With the rapid development of molecular genetics and high-throughput sequencing technologies, candidate-gene-based association analysis has become an important approach for elucidating the genetic basis of economically important traits in pigs. Compared with conventional phenotypic selection, this strategy enables more efficient identification of functional variants affecting growth, reproduction, and meat quality traits [9,10,11,12]. Previous studies have shown that VEGFA polymorphisms are associated with litter size in pigs [13], and that polymorphisms in LTBP2, DLST, and TGFB3 are significantly associated with teat number and body length in American and French Large White pigs [14]. These findings demonstrate the considerable value of the candidate gene approach in pig breeding research. Importantly, linkage disequilibrium analysis revealed that several significant SNPs are in strong LD (C1033T and C1041T with r^2^ = 1.00; G1395A and C1398T with r^2^ ≈ 0.95–0.99), suggesting these associations may reflect the same causal variant or haplotype block rather than multiple independent functional mutations. This highlights the importance of fine-mapping and functional validation to identify the true causal variant(s).

The ARHGEF family encodes Rho guanine nucleotide exchange factors (RhoGEFs), which activate Rho-family GTPases and thereby participate in cytoskeletal remodeling, cell migration, proliferation and differentiation, and tissue morphogenesis. These factors may therefore play important roles in animal growth and development [7,15]. *ARHGEF37* is highly expressed in human hepatocellular carcinoma tissues and is associated with tumor invasion, pulmonary metastasis, and poor prognosis [16]. Mechanistically, *ARHGEF37* functions as a guanine nucleotide exchange factor that directly activates Cdc42, thereby driving cytoskeletal remodeling [16]. Cell-based experiments have further shown that *ARHGEF37* assists dynamin 2 during clathrin-mediated endocytosis, a process critical for cell signaling and membrane trafficking [17]. Therefore, *ARHGEF37* has a biological basis for influencing tissue growth and skeletal muscle development in pigs and represents a candidate functional gene of interest.

The results of this study showed that *ARHGEF37* exhibited a certain degree of genetic polymorphism in both French Large White and French Landrace populations, although their population genetic structures differed markedly between breeds. Most loci in the Large White population conformed to Hardy–Weinberg equilibrium, and PIC values indicated low to moderate polymorphism, suggesting relatively rich genetic variation in the *ARHGEF37* region and its suitability for association analysis. In contrast, the Landrace population showed relatively low overall PIC values, indicating limited genetic variation in this region, possibly because of long-term directional selection and the relatively small population size. These breed differences indicate that the selection potential of the same candidate gene may vary across genetic backgrounds and should be evaluated in the context of population characteristics.

Multiple Testing Correction and Statistical Significance. After applying stringent FDR correction to account for multiple comparisons, none of the associations reached the conventional statistical significance threshold (*q* < 0.05). The lack of statistically significant associations after FDR correction may reflect several factors: (1) the true effects of *ARHGEF37* variants on growth traits in these populations may be small and require larger sample sizes to detect with adequate power; (2) the sample sizes, particularly in Landrace (*n* = 36), may be insufficient to detect moderate genetic effects with stringent significance thresholds; or (3) the examined variants may not be functionally causative. Given these results, the nominally significant associations (uncorrected *p* < 0.05) reported in this study should be considered preliminary and hypothesis-generating rather than definitive evidence of genetic effects. These findings require replication in independent, larger populations before any practical breeding applications.

Single-locus association analysis showed that multiple *ARHGEF37* SNPs showed nominal associations with body weight and chest girth in Large White pigs, though none remained significant after FDR correction. The homozygous mutant genotypes at C1033T, C1041T, G1395A, and C1398T showed more favorable phenotypic values and a pattern consistent with additive gene action. Additive genetic effects are highly valuable in breeding practice because their genetic gains are relatively stable and can be accumulated through continuous selection. These findings suggest that *ARHGEF37* may continuously affect body development by regulating growth-related cellular proliferation and tissue formation. Linkage disequilibrium analysis revealed that C1033T and C1041T are in complete LD (r^2^ = 1.00), indicating they represent a single genetic variant rather than two independent mutations. The identical association patterns observed for these two SNPs are therefore expected and should be interpreted as a single signal. Similarly, G1395A and C1398T are in complete LD (r^2^ = 1.00), representing the same underlying genetic signal.

In the Landrace population, heterozygous genotypes at several loci were associated with greater body weight and chest girth. For example, C1033T, C1041T, C1098T, and T1293C all showed a tendency toward heterozygote advantage. This finding suggests that the genetic effects of *ARHGEF37* in Landrace pigs may be expressed primarily through dominance or overdominance rather than through a purely additive effect. It further indicates that the phenotypic effects of candidate genes are strongly regulated by population genetic background and that the same locus may exhibit different inheritance patterns in different breeds. Linkage disequilibrium analysis revealed that C1033T and C1041T are in complete LD (r^2^ = 1.00), indicating they represent a single genetic variant rather than two independent mutations. The identical association patterns observed for these two SNPs are therefore expected and should be interpreted as a single signal.

Haplotype analysis can further improve the resolution of genetic effects by considering combinations of tightly linked variants. In the present study, haplotype-based association analysis in Large White pigs was restricted to the seven haplotypes meeting the *n* ≥ 5 criterion (H4, H19, H1, H10, H6, H3, and H9), with the results presented in Table 5. Combinations of tightly linked variants may jointly determine gene function and expression, and haplotype analysis may therefore more accurately reflect the genetic effects of a candidate-gene region. Compared with individual loci, favorable haplotypes are stable genetic units and may be more suitable as markers for subsequent MAS applications.

Notably, the Landrace population contained fewer haplotypes, and the limited sample sizes of low-frequency haplotypes reduced the stability of some statistical results. Therefore, some associations identified in Landrace pigs require further validation using larger sample sizes. In particular, the effects of rare genotypes and low-frequency haplotypes may have been overestimated. These effects should be replicated in larger populations and in materials from different generations to improve the reliability of the results.

Overall, this study provides preliminary evidence that *ARHGEF37* may be associated with growth traits in pigs, particularly in French Large White pigs. However, after FDR correction none of the associations remained statistically significant, and the identified alleles and haplotypes should therefore be regarded as candidate markers requiring replication rather than as directly applicable breeding targets. Future studies with larger sample sizes, independent validation cohorts, and functional experiments (e.g., gene expression analysis, protein functional assays, and genome editing) are needed to confirm the effects of *ARHGEF37* on growth traits and to clarify the underlying molecular mechanisms.

It is important to note that association does not prove causation. The SNPs identified here may not be the actual causative mutations but rather markers in linkage disequilibrium with the true functional variant(s). Furthermore, given that no associations survived FDR correction, we cannot establish genuine genetic effects. Definitive establishment of causality requires functional validation studies, including gene expression analysis, protein functional assays, and potentially genome editing approaches.

## 5. Conclusions

This exploratory study examined associations between *ARHGEF37* exon 8 variants and growth traits in French Large White and Landrace pigs. Several nominal associations were observed (e.g., homozygous mutant genotypes at C1033T and C1041T with body weight in Large White pigs; heterozygous genotypes with body weight and chest girth in Landrace pigs), but none survived FDR correction for multiple testing. *ARHGEF37* variants should therefore be considered candidate markers that require replication in larger, independent populations before any application in marker-assisted selection. Because association does not establish causation, functional validation is needed to confirm whether these variants truly affect growth traits.

## 6. Limitations

Several limitations should be acknowledged. First, although FDR correction (Benjamini–Hochberg) was applied across all SNP–trait and haplotype–trait tests, none of the associations remained significant at *q* < 0.05; the nominally significant results reported here are therefore exploratory and require replication. Second, the statistical model did not account for pedigree relationships, batch effects, herd origin, or pen effects because these data were unavailable; a mixed linear model incorporating a kinship matrix would be preferable in future work. Third, despite applying a minimum sample size criterion (*n* ≥ 5), the Landrace population (*n* = 36) and several haplotypes remained small, limiting statistical power and the reliability of effect estimates for low-frequency variants. Fourth, strong linkage disequilibrium between SNP pairs (C1033T–C1041T, r^2^ = 1.00; G1395A–C1398T, r^2^ ≈ 0.95–0.99) indicates that multiple significant associations likely reflect the same genetic signal rather than independent effects. Fifth, formal estimation of additive and dominance effects using quantitative genetics methods was not performed. These limitations indicate that the present findings require validation in larger populations with more rigorous statistical approaches.

## Figures and Tables

**Table 1:** Genotype distribution of *ARHGEF37* SNP loci.

Breed	SNP Locus	Sample Size	Genotype	Genotype Frequency (Number of Individuals)	Allele	Allele Frequency
Large White	C979T	182	CC	0.54 (98)	C	0.73
			CT	0.38 (69)	T	0.27
			TT	0.08 (15)		
	C1033T	182	CC	0.54 (99)	C	0.73
			CT	0.38 (69)	T	0.27
			TT	0.08 (14)		
	C1041T	182	CC	0.54 (99)	C	0.73
			CT	0.38 (69)	T	0.27
			TT	0.08 (14)		
	C1098T	182	CC	0.47 (85)	C	0.66
			CT	0.39 (71)	T	0.34
			TT	0.14 (26)		
	C1262G	182	CC	0.47 (85)	C	0.66
			CG	0.38 (70)	G	0.34
			GG	0.15 (27)		
	T1293C	182	TT	0.65 (119)	T	0.81
			TC	0.31 (56)	C	0.19
			CC	0.04 (7)		
	G1395A	182	GG	0.55 (100)	G	0.74
			GA	0.37 (68)	A	0.26
			AA	0.08 (14)		
	C1398T	182	CC	0.55 (100)	C	0.74
			CT	0.37 (68)	T	0.26
			TT	0.08 (14)		
	G1564C	182	GG	0.51 (93)	G	0.68
			GC	0.35 (63)	C	0.32
			CC	0.14 (26)		
Landrace	C979T	36	CC	0.94 (34)	C	0.97
			CT	0.06 (2)	T	0.03
	C1033T	36	CC	0.67 (24)	C	0.83
			CT	0.33 (12)	T	0.17
	C1041T	36	CC	0.64 (23)	C	0.82
			CT	0.36 (13)	T	0.18
	C1098T	36	CC	0.64 (23)	C	0.81
			CT	0.33 (12)	T	0.19
			TT	0.03 (1)		
	C1262G	36	CC	0.94 (34)	C	0.96
			CG	0.03 (1)	G	0.04
			GG	0.03 (1)		
	T1293C	36	TT	0.67 (24)	T	0.83
			TC	0.33 (12)	C	0.17
	G1395A	36	GG	0.94 (34)	G	0.97
			GA	0.06 (2)	A	0.03
	C1398T	36	CC	0.94 (34)	C	0.97
			CT	0.06 (2)	T	0.03
	G1542A	36	GG	0.72 (26)	G	0.86
			GA	0.28 (10)	A	0.14
	G1564C	36	GG	0.67 (24)	G	0.83
			GC	0.33 (12)	C	0.17

**Table 2:** Genetic diversity analysis of *ARHGEF37* SNP loci.

Breed	SNP Locus	Sample Size	Ho	He	Ne	PIC	X^2^	*p*-Value
Large White	C979T	182	0.60	0.40	1.66	0.32	0.3332	0.5638
	C1033T	182	0.61	0.39	1.64	0.31	0.1666	0.6832
	C1041T	182	0.61	0.39	1.64	0.31	0.1666	0.6832
	C1098T	182	0.55	0.45	1.81	0.35	2.995	0.0835
	C1262G	182	0.55	0.45	1.82	0.35	3.7565	0.0526
	T1293C	182	0.77	0.23	1.30	0.20	0.0164	0.898
	G1395A	182	0.61	0.39	1.63	0.31	0.2646	0.607
	C1398T	182	0.61	0.39	1.63	0.31	0.2646	0.607
	G1564C	182	0.57	0.43	1.76	0.34	7.219	0.0072
Landrace	C979T	36	0.95	0.05	1.06	0.05	0.0294	0.8639
	C1033T	36	0.72	0.28	1.38	0.24	1.44	0.2301
	C1041T	36	0.70	0.30	1.42	0.25	1.7394	0.1872
	C1098T	36	0.69	0.31	1.46	0.26	0.1468	0.7016
	C1262G	36	0.92	0.08	1.09	0.08	15.29	<0.05
	T1293C	36	0.72	0.28	1.38	0.24	1.44	0.2301
	G1395A	36	0.95	0.05	1.06	0.05	0.0294	0.8639
	C1398T	36	0.95	0.05	1.06	0.05	0.0294	0.8639
	G1542A	36	0.76	0.24	1.31	0.21	0.9372	0.333
	G1564C	36	0.72	0.28	1.38	0.24	1.44	0.2301

**Table 3:** Differences in growth traits among *ARHGEF37* SNP genotypes in the Large White population.

Locus	Genotype	Body Weight (kg)	Backfat Thickness (mm)	Loin Muscle Depth (mm)	Body Length (cm)	Chest Girth (cm)	Chest Width (cm)
C979T	CC	96.99 ± 1.02 ^a^	8.21 ± 0.26 ^a^	48.13 ± 0.45 ^a^	111.64 ± 0.75 ^a^	106.41 ± 0.61 ^a^	27.11 ± 0.21 ^a^
	CT	96.48 ± 1.25 ^a^	8.54 ± 0.37 ^a^	47.65 ± 0.67 ^a^	110.33 ± 0.89 ^a^	105.71 ± 0.74 ^a^	27.46 ± 0.24 ^a^
	TT	100.40 ± 3.81 ^a^	8.60 ± 0.73 ^a^	48.24 ± 1.74 ^a^	111.78 ± 1.71 ^a^	107.80 ± 1.82 ^a^	26.87 ± 0.77 ^a^
C1033T	CC	96.80 ± 1.03 ^b^	8.19 ± 0.27 ^a^	48.07 ± 0.46 ^a^	111.56 ± 0.76 ^a^	106.31 ± 0.61 ^a^	27.05 ± 0.22 ^a^
	CT	96.48 ± 1.26 ^b^	8.54 ± 0.38 ^a^	47.66 ± 0.68 ^a^	110.33 ± 0.90 ^a^	105.72 ± 0.75 ^a^	27.46 ± 0.24 ^a^
	TT	102.09 ± 3.68 ^a^	8.84 ± 0.74 ^a^	48.74 ± 1.80 ^a^	112.39 ± 1.73 ^a^	108.66 ± 1.74 ^a^	27.35 ± 0.66 ^a^
C1041T	CC	96.80 ± 1.03 ^b^	8.19 ± 0.27 ^a^	48.07 ± 0.46 ^a^	111.56 ± 0.76 ^a^	106.31 ± 0.61 ^a^	27.05 ± 0.22 ^a^
	CT	96.48 ± 1.26 ^b^	8.54 ± 0.38 ^a^	47.66 ± 0.68 ^a^	110.33 ± 0.90 ^a^	105.72 ± 0.75 ^a^	27.46 ± 0.24 ^a^
	TT	102.09 ± 3.68 ^a^	8.84 ± 0.74 ^a^	48.74 ± 1.80 ^a^	112.39 ± 1.73 ^a^	108.66 ± 1.74 ^a^	27.35 ± 0.66 ^a^
C1098T	CC	96.75 ± 1.10 ^a^	8.15 ± 0.28 ^a^	48.11 ± 0.50 ^a^	111.32 ± 0.83 ^a^	106.39 ± 0.65 ^a^	27.09 ± 0.24 ^a^
	CT	96.68 ± 1.32 ^a^	8.75 ± 0.39 ^a^	47.65 ± 0.61 ^a^	110.73 ± 0.90 ^a^	105.77 ± 0.74 ^a^	27.25 ± 0.23 ^a^
	TT	99.27 ± 2.24 ^a^	8.07 ± 0.51 ^a^	48.35 ± 1.33 ^a^	111.77 ± 1.26 ^a^	107.20 ± 1.36 ^a^	27.64 ± 0.45 ^a^
C1262G	CC	96.75 ± 1.10 ^a^	8.15 ± 0.28 ^a^	48.11 ± 0.50 ^a^	111.32 ± 0.83 ^a^	106.39 ± 0.65 ^a^	27.09 ± 0.24 ^a^
	CG	96.68 ± 1.34 ^a^	8.79 ± 0.39 ^a^	47.67 ± 0.62 ^a^	110.64 ± 0.91 ^a^	105.75 ± 0.75 ^a^	27.29 ± 0.23 ^a^
	GG	99.18 ± 2.15 ^a^	7.98 ± 0.50 ^a^	48.27 ± 1.29 ^a^	111.96 ± 1.23 ^a^	107.19 ± 1.31 ^a^	27.50 ± 0.46 ^a^
T1293C	CC	97.53 ± 4.55 ^a^	8.06 ± 1.10 ^a^	49.79 ± 1.03 ^a^	112.06 ± 2.66 ^a^	107.34 ± 2.26 ^a^	27.24 ± 0.87 ^a^
	TC	96.61 ± 1.33 ^a^	7.95 ± 0.37 ^a^	47.54 ± 0.63 ^a^	111.09 ± 0.88 ^a^	105.69 ± 0.86 ^a^	27.11 ± 0.27 ^a^
	TT	97.28 ± 1.01 ^a^	8.59 ± 0.27 ^a^	48.06 ± 0.50 ^a^	111.13 ± 0.72 ^a^	106.47 ± 0.56 ^a^	27.28 ± 0.20 ^a^
G1395A	AA	102.09 ± 3.68 ^a^	8.84 ± 0.74 ^a^	48.74 ± 1.80 ^a^	112.39 ± 1.73 ^a^	108.66 ± 1.74 ^a^	27.35 ± 0.66 ^a^
	GA	96.39 ± 1.27 ^b^	8.39 ± 0.35 ^a^	47.76 ± 0.68 ^a^	110.28 ± 0.91 ^a^	105.57 ± 0.75 ^b^	27.44 ± 0.24 ^a^
	GG	96.86 ± 1.03 ^b^	8.29 ± 0.29 ^a^	48.00 ± 0.46 ^a^	111.58 ± 0.75 ^a^	106.40 ± 0.61 ^ab^	27.07 ± 0.22 ^a^
C1398T	CC	96.86 ± 1.03 ^b^	8.29 ± 0.29 ^a^	48.00 ± 0.46 ^a^	111.58 ± 0.75 ^a^	106.40 ± 0.61 ^ab^	27.07 ± 0.22 ^a^
	CT	96.39 ± 1.27 ^b^	8.39 ± 0.35 ^a^	47.76 ± 0.68 ^a^	110.28 ± 0.91 ^a^	105.57 ± 0.75 ^b^	27.44 ± 0.24 ^a^
	TT	102.09 ± 3.68 ^a^	8.84 ± 0.74 ^a^	48.74 ± 1.80 ^a^	112.39 ± 1.73 ^a^	108.66 ± 1.74 ^a^	27.35 ± 0.66 ^a^
G1564C	CC	99.27 ± 2.24 ^a^	8.07 ± 0.51 ^a^	48.35 ± 1.33 ^a^	111.77 ± 1.26 ^a^	107.20 ± 1.36 ^a^	27.64 ± 0.45 ^a^
	GC	95.43 ± 1.37 ^b^	8.54 ± 0.38 ^a^	47.23 ± 0.65 ^a^	110.03 ± 0.94 ^a^	105.27 ± 0.78 ^a^	27.21 ± 0.26 ^a^
	GG	97.59 ± 1.07 ^ab^	8.34 ± 0.29 ^a^	48.35 ± 0.47 ^a^	111.75 ± 0.79 ^a^	106.68 ± 0.62 ^a^	27.13 ± 0.23 ^a^

Note: Data are presented as mean ± SE. Within the same locus, values with different superscript letters differ significantly (*p* < 0.05).

**Table 4:** Differences in growth traits among *ARHGEF37* SNP genotypes in the Landrace population.

Locus	Genotype	Body Weight (kg)	Backfat Thickness (mm)	Loin Muscle Depth (mm)	Body Length (cm)	Chest Girth (cm)	Chest Width (cm)
C979T	CC	97.33 ± 2.30 ^a^	8.72 ± 0.44 ^a^	45.51 ± 0.83 ^a^	113.62 ± 1.28 ^a^	105.12 ± 1.33 ^a^	25.99 ± 0.40 ^a^
	CT	102.40 ± 10.60 ^a^	7.40 ± 0.50 ^a^	45.25 ± 1.35 ^a^	115.55 ± 5.15 ^a^	108.45 ± 1.05 ^a^	25.20 ± 2.30 ^a^
C1033T	CC	95.59 ± 2.88 ^b^	8.40 ± 0.58 ^a^	45.38 ± 0.90 ^a^	112.85 ± 1.57 ^a^	104.38 ± 1.70 ^b^	25.64 ± 0.41 ^a^
	CT	101.68 ± 3.2 ^a^	9.18 ± 0.52 ^a^	45.74 ± 1.58 ^a^	115.49 ± 1.93 ^a^	107.16 ± 1.69 ^a^	26.56 ± 0.83 ^a^
C1041T	CC	95.03 ± 2.95 ^b^	8.23 ± 0.58 ^a^	45.56 ± 0.93 ^a^	112.87 ± 1.64 ^a^	104.01 ± 1.73 ^b^	25.62 ± 0.43 ^a^
	CT	102.19 ± 2.99 ^a^	9.41 ± 0.53 ^a^	45.40 ± 1.49 ^a^	115.26 ± 1.79 ^a^	107.60 ± 1.62 ^a^	26.52 ± 0.77 ^a^
C1098T	CC	95.03 ± 2.95 ^b^	8.23 ± 0.58 ^a^	45.56 ± 0.93 ^a^	112.87 ± 1.64 ^a^	104.01 ± 1.73 ^b^	25.62 ± 0.43 ^a^
	CT	103.06 ± 3.11 ^a^	9.62 ± 0.53 ^a^	45.53 ± 1.62 ^a^	115.67 ± 1.89 ^a^	107.62 ± 1.76 ^a^	26.83 ± 0.77 ^a^
	TT	91.8 ± 0.00	6.9 ± 0.00	43.9 ± 0.00	110.40 ± 0.00	107.40 ± 0.00	22.90 ± 0.00
C1262G	CC	97.34 ± 2.30	8.73 ± 0.44	45.52 ± 0.83	113.62 ± 1.28	105.12 ± 1.34	25.99 ± 0.40
	CG	113.00 ± 0.00	7.9 ± 0.00	46.60 ± 0.00	120.70 ± 0.00	109.50 ± 0.00	27.50 ± 0.00
	GG	91.80 ± 0.00	6.9 ± 0.00	43.90 ± 0.00	110.40 ± 0.00	107.40 ± 0.00	22.90 ± 0.00
T1293C	TC	101.29 ± 3.10 ^a^	9.53 ± 0.56 ^a^	45.30 ± 1.62 ^a^	114.81 ± 1.88 ^a^	107.44 ± 1.75 ^a^	26.44 ± 0.83 ^a^
	TT	95.78 ± 2.92 ^b^	8.22 ± 0.55 ^a^	45.60 ± 0.89 ^a^	113.19 ± 1.61 ^a^	104.24 ± 1.67 ^b^	25.70 ± 0.42 ^a^
G1395A	GA	102.4 ± 10.6 ^a^	7.40 ± 0.50 ^a^	45.25 ± 1.35 ^a^	115.55 ± 5.15 ^a^	108.45 ± 1.05 ^a^	25.20 ± 2.30 ^a^
	GG	97.34 ± 2.30 ^a^	8.73 ± 0.44 ^a^	45.52 ± 0.83 ^a^	113.62 ± 1.28 ^a^	105.12 ± 1.34 ^b^	25.99 ± 0.40 ^a^
C1398T	CC	97.34 ± 2.30 ^a^	8.73 ± 0.44 ^a^	45.52 ± 0.83 ^a^	113.62 ± 1.28 ^a^	105.12 ± 1.34 ^b^	25.99 ± 0.40 ^a^
	CT	102.40 ± 10.60 ^a^	7.40 ± 0.50 ^a^	45.25 ± 1.35 ^a^	115.55 ± 5.15 ^a^	108.45 ± 1.05 ^a^	25.20 ± 2.30 ^a^
G1542A	GA	101.19 ± 3.37 ^a^	10.01 ± 0.67 ^a^	44.89 ± 1.86 ^a^	116.36 ± 2.15 ^a^	106.74 ± 2.03 ^a^	27.41 ± 0.70 ^a^
	GG	96.24 ± 2.78 ^a^	8.14 ± 0.49 ^b^	45.74 ± 0.85 ^a^	112.72 ± 1.47 ^a^	104.75 ± 1.58 ^a^	25.39 ± 0.43 ^a^
G1564C	GC	102.43 ± 2.96 ^a^	9.75 ± 0.58 ^a^	45.22 ± 1.55 ^a^	116.32 ± 1.87 ^a^	106.93 ± 1.69 ^a^	27.48 ± 0.58 ^a^
	GG	95.21 ± 2.91 ^b^	8.11 ± 0.53 ^a^	45.65 ± 0.91 ^a^	112.44 ± 1.55 ^a^	104.49 ± 1.70 ^a^	25.18 ± 0.44 ^a^

Note: Data are presented as mean ± SE. Within the same locus, values with different superscript letters differ significantly (*p* < 0.05).

**Table 5:** Differences in growth traits among *ARHGEF37* haplotypes in the pig populations.

Breed	Haplotype	Body Weight (kg)	Backfat Thickness (mm)	Loin Muscle Depth (mm)	Body Length (cm)	Chest Girth (cm)	Chest Width (cm)
Large White pigs	H1	95.21 ± 4.68 ^abcd^	7.39 ± 0.70 ^bc^	47.74 ± 2.06 ^abc^	110.35 ± 2.13 ^abc^	105.93 ± 2.48 ^abc^	26.94 ± 0.91 ^a^
	H2	109.15 ± 4.65 ^a^	9.05 ± 1.05 ^bc^	48.95 ± 1.85 ^abc^	109.10 ± 2.10 ^abc^	109.45 ± 1.05 ^bc^	28.85 ± 1.15 ^a^
	H3	105.24 ± 3.02 ^ab^	10.88 ± 2.26 ^ab^	52.82 ± 1.14 ^a^	119.02 ± 3.11 ^a^	107.18 ± 1.96 ^abc^	27.62 ± 0.84 ^a^
	H4	96.98 ± 1.14 ^abcd^	8.19 ± 0.29 ^bc^	48.17 ± 0.52 ^abc^	111.39 ± 0.89 ^abc^	106.49 ± 0.68 ^abc^	27.08 ± 0.25 ^a^
	H5	101.00 ± 0.90 ^abcd^	14.40 ± 0.60 ^a^	50.65 ± 1.25 ^abc^	114.55 ± 6.15 ^ab^	108.55 ± 2.95 ^abc^	26.75 ± 1.35 ^a^
	H6	95.00 ± 3.52 ^abcd^	7.44 ± 0.88 ^bc^	47.46 ± 1.62 ^abc^	111.34 ± 1.91 ^abc^	105.23 ± 2.80 ^abc^	26.56 ± 0.61 ^a^
	H7	92.00 ± 15.10 ^bcd^	5.00 ± 1.30 ^c^	44.50 ± 5.30 ^abc^	106.85 ± 6.85 ^abc^	98.50 ± 5.90 ^c^	26.60 ± 3.60 ^a^
	H8	101.08 ± 6.97 ^abcd^	9.75 ± 2.52 ^abc^	49.23 ± 0.92 ^abc^	113.98 ± 3.74 ^ab^	110.90 ± 2.79 ^a^	28.88 ± 0.86 ^a^
	H9	90.74 ± 3.95 ^bcd^	9.02 ± 1.46 ^bc^	44.54 ± 1.66 ^abc^	105.78 ± 3.63 ^bc^	102.42 ± 3.56 ^abc^	26.44 ± 0.80 ^a^
	H10	97.94 ± 2.91 ^abcd^	9.69 ± 1.75 ^abc^	49.31 ± 3.09 ^abc^	111.83 ± 1.98 ^abc^	108.71 ± 3.12 ^abc^	27.63 ± 0.58 ^a^
	H11	100.60 ± 7.50 ^abcd^	8.20 ± 2.40 ^bc^	48.20 ± 5.30 ^abc^	114.15 ± 4.85 ^ab^	103.75 ± 4.25 ^abc^	27.60 ± 1.10 ^a^
	H12	101.30 ± 3.43 ^abcd^	7.58 ± 1.19 ^bc^	46.70 ± 1.37 ^abc^	113.23 ± 2.89 ^abc^	105.20 ± 1.62 ^abc^	26.60 ± 1.42 ^a^
	H13	86.60 ± 16.30 ^d^	9.25 ± 4.05 ^bc^	41.30 ± 7.90 ^c^	100.15 ± 8.15 ^c^	100.15 ± 6.55 ^bc^	27.00 ± 2.70 ^a^
	H14	87.10 ± 4.36 ^cd^	7.43 ± 1.24 ^bc^	42.87 ± 2.45 ^bc^	107.77 ± 2.54 ^abc^	102.47 ± 1.21 ^abc^	26.33 ± 0.99 ^a^
	H15	95.73 ± 7.83 ^abcd^	7.40 ± 0.82 ^bc^	47.10 ± 3.70 ^abc^	105.00 ± 0.98 ^bc^	108.87 ± 4.70 ^abc^	27.03 ± 1.74 ^a^
	H16	99.93 ± 6.72 ^abcd^	8.47 ± 1.07 ^bc^	44.67 ± 0.17 ^abc^	111.07 ± 0.97 ^abc^	105.63 ± 3.52 ^abc^	28.30 ± 1.11 ^a^
	H17	101.90 ± 15.60 ^abc^	10.30 ± 2.90 ^ab^	45.00 ± 1.60 ^abc^	102.10 ± 4.50 ^bc^	104.55 ± 3.55 ^abc^	29.60 ± 1.20 ^a^
	H18	98.73 ± 6.92 ^abcd^	9.13 ± 1.16 ^bc^	52.03 ± 2.08 ^ab^	109.73 ± 6.43 ^abc^	107.63 ± 4.97 ^abc^	28.78 ± 0.66 ^a^
	H19	99.87 ± 2.90 ^abcd^	8.30 ± 0.63 ^bc^	50.41 ± 1.56 ^abc^	111.20 ± 1.57 ^abc^	107.78 ± 1.50 ^abc^	27.72 ± 0.57 ^a^
Landrace pigs	H1	95.03 ± 2.95 ^ab^	8.23 ± 0.58 ^a^	45.56 ± 0.93 ^a^	112.87 ± 1.64 ^ab^	104.01 ± 1.73 ^a^	25.62 ± 0.43 ^a^
	H2	103.33 ± 3.29 ^a^	9.17 ± 0.78 ^a^	45.37 ± 3.79 ^a^	119.60 ± 1.98 ^a^	108.37 ± 2.33 ^a^	26.83 ± 1.47 ^a^
	H3	89.50 ± 14.8 ^b^	7.40 ± 1.60 ^a^	41.55 ± 5.55 ^a^	105.35 ± 6.15 ^b^	100.00 ± 6.30 ^a^	26.15 ± 2.05 ^a^

Note: Haplotypes with *n* < 5 are listed for completeness; the superscript letters for these low-frequency haplotypes should be interpreted with caution due to the small sample sizes. Data are presented as mean ± SE. Within the same population, values with different superscript letters differ significantly (*p* < 0.05).

## Data Availability

The original contributions presented in this study are included in the article/Supplementary Materials. Further inquiries can be directed to the corresponding author.

## Supplementary Materials

The following supporting information can be downloaded at: https://www.mdpi.com/article/10.3390/ani16213411/s1, Table S1: FDR-corrected *p*-values and *q*-values for all association tests; Table S2: Linkage disequilibrium (LD); Table S3: Raw genotype data at ten SNP loci in *ARHGEF37*; Table S4: Phenotypic and genotypic data with population genetic parameters.
